# Alpha and theta oscillations are inversely related to progressive levels of meditation depth

**DOI:** 10.1093/nc/niab042

**Published:** 2021-11-29

**Authors:** Sucharit Katyal, Philippe Goldin

**Affiliations:** Betty Irene Moore School of Nursing, University of California Davis Medical Center, Sacramento, CA 95817, California; Betty Irene Moore School of Nursing, University of California Davis Medical Center, Sacramento, CA 95817, California

**Keywords:** attention, consciousness, mindfulness, meditation, EEG, psychophysiology

## Abstract

Meditation training is proposed to enhance mental well-being by modulating neural activity, particularly alpha and theta brain oscillations, and autonomic activity. Although such enhancement also depends on the quality of meditation, little is known about how these neural and physiological changes relate to meditation quality. One model characterizes meditation quality as five increasing levels of ‘depth’: hindrances, relaxation, concentration, transpersonal qualities and nonduality. We investigated the neural oscillatory (theta, alpha, beta and gamma) and physiological (respiration rate, heart rate and heart rate variability) correlates of the self-reported meditation depth in long-term meditators (LTMs) and meditation-naïve controls (CTLs). To determine the neural and physiological correlates of meditation depth, we modelled the change in the slope of the relationship between self-reported experiential degree at each of the five depth levels and the multiple neural and physiological measures. CTLs reported experiencing more ‘hindrances’ than LTMs, while LTMs reported more ‘transpersonal qualities’ and ‘nonduality’ compared to CTLs, confirming the experiential manipulation of meditation depth. We found that in both groups, theta (4–6 Hz) and alpha (7–13 Hz) oscillations were related to meditation depth in a precisely opposite manner. The theta amplitude positively correlated with ‘hindrances’ and increasingly negatively correlated with increasing meditation depth levels. Alpha amplitude negatively correlated with ‘hindrances’ and increasingly positively with increasing depth levels. The increase in the inverse association between theta and meditation depth occurred over different scalp locations in the two groups—frontal midline in LTMs and frontal lateral in CTLs—possibly reflecting the downregulation of two different aspects of executive processing—monitoring and attention regulation, respectively—during deep meditation. These results suggest a functional dissociation of the two classical neural signatures of meditation training, namely, alpha and theta oscillations. Moreover, while essential for overcoming ‘hindrances’, executive neural processing appears to be downregulated during deeper meditation experiences.

**Highlights**

- Our study reveals neurophysiological changes that occur as meditation experiences become deeper.
- Alpha and theta brainwaves are two reliable neurophysiological signatures of meditation.
- Theta activity increased with more distractions and was suppressed during deeper experiences.
- Increased alpha activity was related to fewer distractions and more deeper meditation experiences.
- Deeper meditation experiences appear to involve a suppression of executive neural processing.

## Introduction

Meditation training has been shown to improve executive functioning and mental health (Chiesa and Serretti 2009; Leyland *et al.* 2019). These psychological changes are accompanied by reliable changes in brain (Cahn and Polich 2006; Lomas *et al.* 2015) and autonomic (Jevning *et al.* 1992) activity. However, little is known about how these neural physiological changes are related to the quality or ‘depth’ of meditation (Wallace 1999; Lutz *et al.* 2007; Dunne *et al.* 2019). Such an understanding is essential for unravelling how meditation training impacts psychological well-being (Brown *et al.* 2007; Lutz *et al.* 2015).

The study of subjective experience during meditation requires overcoming reliability issues pertaining to introspective reports. This could be done through a Husserlian epoché-and-reduction approach to obtaining generic structures of subjective experience and correlating them with neurobiological measurements—a paradigm known as neurophenomenology (Varela 1996). Or it could be done through a ‘front-loading’ approach, where prior phenomenological insight about an experimental procedure is used to obtain experiential self-reports (Gallagher and Sørensen 2006).

Berkovich-Ohana and colleagues used magnetoencephalography (MEG) to study brain correlates of ‘self-dissolution’ during meditation using both a front-loaded paradigm (Dor-Ziderman *et al.* 2013) and a neurophenomenological approach (Dor-Ziderman *et al.* 2016). They found that heightened self-dissolution was related to decreased beta-band (13–25 Hz) activity in the parietal cortex. Another study periodically probed participants to determine whether they were ‘on task’ or ‘mind-wandering’ during focused attention meditation and found an increase in alpha (7–13 Hz) and frontal midline theta (4–6 Hz) oscillations when they reported being on task versus mind-wandering (Brandmeyer and Delorme 2018). Their findings support a large body of literature showing a reliable increase in alpha and theta oscillations during meditation practice, irrespective of the type of meditation practiced or amount of training (Cahn and Polich 2006; Lomas *et al.* 2015). However, it was unclear how these oscillations were related to the *way* meditators were on task, for example, effortfully or effortlessly (Lutz *et al.* 2015). Effortful and effortless concentration during meditation was examined in another study, which found that deactivation of gamma-band (30–50 Hz) EEG activity in the posterior cingulate cortex corresponded to the experience of effortless concentration in both trained and novice meditators (van Lutterveld *et al.* 2017). Current evidence thus suggests that introspection-related brain activity during meditation can be observed in theta, alpha, beta and gamma oscillatory frequency bands.

Beyond measuring meditation depth along specific experiential dimensions like self-dissolution and effortless concentration, researchers have constructed a more general classification system in which different experiences are grouped into levels of meditation depth (Piron 2001). Assessing advanced meditators from a variety of contemplative traditions, Piron (2001) developed Meditation Depth Questionnaire (MEDEQ) to probe meditation quality independent of meditation tradition or type. The MEDEQ characterizes depth in five progressive levels. The first level, *hindrances*, includes challenges associated with practicing meditation, such as drowsiness and distraction. The second level captures experiences of *relaxation*. The third level captures experiences such as effortless *concentration*. The fourth level, *transpersonal qualities*, captures interpersonal and positive affect experiences, which occur more consistently at advanced stages of training. Finally, the fifth level characterizes experiences of *nonduality* such as reduced subject–object distinction, self-dissolution, which usually require extensive training to master (Piron 2001; Josipovic 2014). The MEDEQ thus provides a front-loading phenomenological instrument for investigating the neurobiological substrates of meditation depth.

Meditation practice also alters autonomic activity, specifically, decreasing respiration rate (RR) (Corby *et al.* 1978; Wielgosz *et al.* 2016) and increasing heart rate variability (HRV) (Lehrer *et al.* 1999; Sarang and Telles 2006; Tang *et al.* 2009). Additionally, decreased heart rate (HR) has also been observed during meditation (Delmonte 1985; Zeidan *et al.* 2010), although practices engaging the affective system (e.g., compassion for others) are accompanied by increased HR following long-term training (Lutz *et al.* 2009; Lumma *et al.* 2015). Interoceptive signals play an important role in theories of conscious experience, especially pertaining to self and affect (Damasio 1999; Gallagher 2005; Seth 2013). However, the relationship between autonomic signals and meditation experience is yet to be carefully examined.

We investigated whether the self-reported meditation depth was associated with seven neuro-physiological measures (NPMs); four neural oscillatory bands (theta, alpha, beta and gamma) and three autonomic measures (RR, HR and HRV) in long-term meditators (LTMs) and demographically matched meditation-naïve control participants (CTL). To induce variability in meditation depth, we administered an abbreviated version of the MEDEQ immediately following four different conditions: listening to a story as baseline (BL), listening to chanting (CH) and two different meditation practices (M1 and M2). For M1 and M2, the CTLs engaged in *mindfulness of breath* and *loving-kindness meditation* practices widely used in mindfulness-based interventions, and the LTMs engaged in their daily practices of *mantra concentration* and *nonduality* (see Methods). Because the MEDEQ was developed to measure specific subjective experiences during meditation practice irrespective of meditation type or tradition, it allows us to combine or compare LTMs and CTLs despite implementation of different meditation practices during M1 and M2.

We expected that, compared to CTLs, LTMs would experience greater meditation depth (Hypothesis 1). Specifically, we expected fewer *hindrances* and greater *concentration, transpersonal qualities* and *nonduality* following M1 and M2 in LTMs versus CTLs and greater *relaxation* in both groups following CH, M1 and M2 versus BL. As introspection-related brain activity has previously been observed in multiple frequency bands, including theta, alpha, beta and gamma, our hypothesis for the neural correlates of meditation was relatively broad and included all four of these bands. We expected that across participants a greater meditation depth would be positively associated with alpha, theta, gamma and HRV and negatively associated with beta, RR HR (Hypothesis 2). Because meditation depth was obtained in five levels, we measured this association as a change in slope across the five depth levels in the correlation between self-reported ratings at a particular depth level and an NPM. A significant increase in the slope across increasing depth levels would indicate a positive association and a decrease in the slope would indicate a negative association. Finally, it is possible that the two groups use different neurobiological strategies to provide self-reports. We therefore also expected that the association between NPMs and meditation depth would be significantly different between groups (Hypothesis 2a).

## Materials and methods

### Participants

We recruited LTMs (*n* = 13; 4 females; mean ± SD meditation training: 32.2 ± 9.7 years) and meditation-naïve CTLs (*n *= 15; 6 females). The groups did not differ significantly on age (LTM = 56.8 ± 12.3; CTL = 53.5 ± 14.2 years; Wilcoxon rank-sum test, *P *= 0.83) and years of formal education (LTM = 15.9 ± 3.0; CTL = 19.0 ± 1.2; *P *= 0.08). Participants reported no psychiatric or neurological diagnoses. All participants provided written informed consent in compliance with the Institutional Review Board of the University of California Davis.

### EEG and physiological data acquisition

We used a 32-channel BrainVision ActiChamp system (Brain Products, Germany) to measure continuous EEG activity (sampling frequency 1000 Hz). The active ActiChamp electrodes help improve the signal-to-noise ratio. Each channel was ensured to have an impedance below 16 kΩ prior to data collection. We also measured continuous respiration and pulse (sampling frequency 128 Hz) using a respiration belt and a photoplethysmogram, respectively, through a NeXus-10 (Mind Media, Netherlands) device.

### Procedure

Before beginning measurements, participants familiarized themselves with the MEDEQ questions to ensure they understood the meaning of each question. The participants first underwent a task measuring self-referential processing published elsewhere (Katyal *et al.* 2020). This was followed by four blocks (i) listening to a podcast story (BL; 9 min), (ii) listening to CH music (CH; 6 min), (iii) meditation 1 (M1; 20 min) and (iv) meditation 2 (M2; 15 min). See Supplementary Materials for details about BL, CH, M1 and M2.

### Self-reported meditation depth

We assessed self-reported meditation depth immediately after each block. To reduce participant burden, we identified a subset of items (14 out of 30) that assessed each of the five depth levels, specifically, MEDEQ items 2, 4, 6, 8, 10, 11, 12, 15, 19, 22, 23, 24, 26 and 27 (Piron 2001). We labeled the five depth levels DL0 to DL4. Because *hindrances* are inversely related to the other depth levels, it was labelled DL0. The participants were required to rate each item using a Likert scale from 0 (not at all) to 6 (medium) to 12 (maximum).

### Statistical analyses

We used linear mixed models (LMMs) as implemented by the *lme4* package (version 1.1.21) in R (Bates *et al.* 2014) for all analyses. The intercept for individual participants was used as the random effect for all LMMs. Even though the self-reported meditation depth data was based on a Likert scale, which is ordinal, using more than four levels (we used a 12-point scale for each item of the MEDEQ) allows its treatment as continuous data (Johnson and Creech 1983; Norman 2010). LMMs were examined to ensure they satisfied assumptions of linearity, homoscedasticity and normality of residuals. Interaction and main effects from the LMMs were evaluated using type II Wald *χ*^2^ tests through the *Anova* function in the *car* package (version 3.0.2). Post-hoc differences were evaluated using *lsmeans* function, which uses the Kenward–Roger method for estimating the degrees of freedom and performs a multiple comparison correction using the Tukey method for a family of four estimates.

#### Self-reported rating at each depth level

Participants provided Likert-scale ratings for MEDEQ questions that loaded on each of the five depth levels (DL0–DL4). For Hypothesis 1, we investigated the two-way interaction and main effects of *block* (BL, CH, M1, M2) by *group* (LTM, CTL) on the ratings at each depth level.

#### Association between meditation depth and neurophysiological measures

For an NPM to be associated with meditation depth (Hypothesis 2), there would have to be a significant change in the correlation between the NPM and self-reported rating across the five meditation depth levels. For example, if the strength of alpha oscillations is positively associated with meditation depth, then the correlation between alpha and self-reported rating across the four listening/meditation blocks would be low (or even negative) for DL0 (*hindrances*) and would significantly increase up to DL4 (*nonduality*). Such an effect can be statistically determined as a two-way interaction between alpha amplitude and depth level (in its five categorical levels, DL0 to DL4) when regressed upon the self-reported ratings, which allows us to infer if the slope of the regression of alpha amplitude upon the ratings changes significantly across the five levels of meditation depth. This kind of mixed modelling approach is akin to performing a repeated measures analysis of covariance.

Moreover, if a particular NPM was related to meditation depth differently between the two groups (Hypothesis 2a), we would observe a three-way interaction between NPM, depth level and group when regressed upon self-reported ratings.

It is possible that some of the NPMs are correlated among themselves (Abdullah *et al.* 2010) and their association with meditation depth may be mediated by another NPM. To avoid this possibility and to obtain the NPMs that best independently predicted meditation depth, we constructed a single multilinear mixed model where seven three-way interactions (NPM × depth level × group) and seven two-way interactions (NPM × depth level) were regressed upon self-reported ratings. For evaluating the three- and two-way interactions, their dependent two-way interactions (NPM × group and group × depth level) and main effects (all NPMs, group, depth level) were also included in the model.

To obtain the NPMs that were significantly associated with meditation depth, we used a stepwise top-down model reduction approach. For this, we first evaluated the full model using maximum likelihood estimation (Zuur *et al.* 2009). Then, in a stepwise manner, we first reduced the three-way interactions and then the two-way interactions that did not contribute significantly to the model. For model reduction, at each step we selected the interaction with the lowest *χ*^2^ value (based on a Type II Wald *χ*^2^ test) and evaluated if it contributed to the model significantly (alpha = 0.05; Bonferroni corrected for 14 comparisons, seven two- and seven three-way interactions each) using the likelihood ratio test in the *anova* function in the R *stats* package (version 3.4.4). If the interaction did not contribute significantly to the model, it was removed. When removing the three-way interaction, we also removed its dependent two-way interactions (i.e., NPM × group). Similarly, when removing a two-way interaction, we also removed the corresponding main effect. This procedure was followed until only the significant interactions remained. We finally evaluated this reduced model with restricted maximum likelihood (REML) estimation to obtain accurate *P*-values (Zuur *et al.* 2009).

Post-hoc analysis consisted of evaluating the association between the significant NPM and self-reports at each depth level separately. This was done using *t*-tests with Satterthwaite’s method to estimate the degrees of freedom through the *summary* function of the *lmerTest* package (version 3.0.1). The *P*-values for the five depth levels were multiple comparisons corrected using false discovery rate.

## Results

Hypothesis 1: Self-reported ratings at different levels of meditation depth

**Figure 1.**
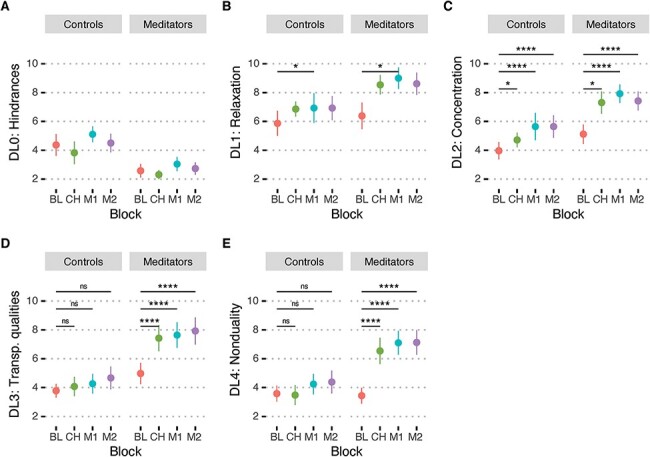
Self-reports of meditation depth for the five depth levels: (A) hindrances (DL0), (B) relaxation (DL1), (C) concentration (DL2), (D) transpersonal qualities (DL3) and (E) nonduality (DL4). Each depth level is plotted for the two groups, controls and long-term meditators, and for four time-points, baseline (BL), chanting (CH), first meditation practice (M1) and second meditation practice (M2). The error bars indicate standard errors of the means. Significance levels: *P < 0.05; **P < 0.01; *P* < 0.005; ****P < 0.001

Figure 1 shows ratings for the five depth levels of the MEDEQ for the two groups (LTMs, CTLs) and four blocks (BL, CH, M1, M2). We first tested for between-group differences in the five depth levels at BL as an indicator of trait differences. While there was a trend for LTMs to have fewer hindrances (DL0) than CTLs during BL (*t*(26) = −1.95; *P* = 0.063), none of the other depth levels were different between groups (DL1: *t*(26) = 0.41; *P* = 0.69; DL2: *t*(26) = 1.28; *P* = 0.21; DL3: *t*(26) = 1.41; *P* = 0.17; DL4: *t*(26) = −0.18; *P* = 0.86).

Hypothesis 2: Association between self-reports and neuro-physiology

Table 1 shows the interaction and main effects for the different depth levels along with the hypothesized post-hoc comparisons (post-hoc *P*-values adjusted using Tukey method for a family of four estimates). For *hindrances* (DL0), there was a significant main effect of group, with CTLs reporting significantly greater *hindrances* than the LTMs. For *relaxation* (DL1), there was a significant effect of block, with significantly greater *relaxation* during M1 compared to BL. For *concentration* (DL2), there were significant main effects of block, with significantly greater reports of *concentration* for CH, M1 and M2 compared to BL, and group with significantly greater *concentration* for LTMs compared to CTLs. For *transpersonal qualities* (DL3), there was a significant group by block interaction characterized by increased ratings of *transpersonal qualities* during CH, M1 and M2 compared to BL for the LTMs versus CTLs. For *nonduality* (DL4), there was a significant group by block interaction characterized by greater self-reports of *nonduality* for CH, M1 and M2 compared to BL for LTMs versus CTLs.

**Table 1.** Statistical effects for the linear mixed models corresponding to the five depth levels

	Interaction		Main effect		Main effect		
	group × block		group		block		
Depth level	*χ* ^2^(3)	*P*	*χ* ^2^(1)	*P*	*χ* ^2^(3)	*P*	Post-hoc comparisons
Hindrances	0.43	0.94	6.68	0.010	6.67	0.083	CTL > LTM *t*(78.38) = 2.81; *P* = 0.031
Relaxation	1.86	0.60	3.23	0.073	10.35	0.016	M1 > BL *t*(78.38) = 2.81; *P* = 0.031
Concentration	2.14	0.55	5.86	0.016	24.36	<0.001	CH > BL *t*(78.49) = 2.96; *P* = 0.021 M1 > BL *t*(78.66) = 4.48; *P* < 0.001 M2 > BL *t*(78.77) = 4.00; *P *< 0.001
Transpersonal qualities	9.19	0.027					LTM: CH > BL *t*(75.20) = 4.00; *P* < 0.001 M1 > BL *t*(75.20) = 4.36; *P* < 0.001 M2 > BL *t*(75.20) = 4.82; *P *< 0.001 CTL: CH > BL *t*(75.42) = 0.71; *P* = 0.89 M1 > BL *t*(75.63) = 0.86; *P* = 0.82 M2 > BL *t*(75.63) = 1.56; *P* = 0.41
Nonduality	21.46	<0.001					LTM: CH > BL *t*(75.10) = 5.25; *P* < 0.001 M1 > BL *t*(75.10) = 6.20; *P* < 0.001 M2 > BL *t*(75.10) = 6.24; *P *< 0.001 CTL: CH > BL *t*(75.32) =-0.01; *P* = 1.00 M1 > BL *t*(75.53) = 0.97; *P* = 0.77 M2 > BL *t*(75.53) = 1.23; *P* = 0.61

**Figure 2.**
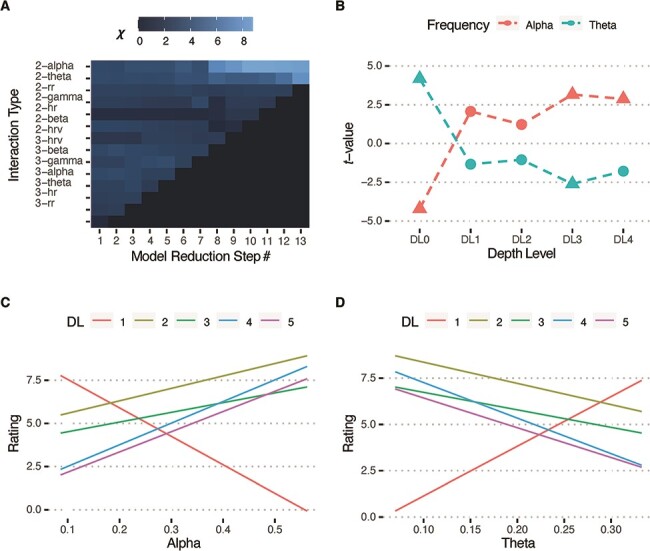
(A) Stepwise model reduction. Columns show the steps and rows show the three- and two-way interactions modelled at that step. The square-root of the *χ*^2^ value of the interaction is shown in shades of grey. Step 1 included all interactions. At each step the weakest interaction was dropped. The final reduced model revealed two significant two-way interactions; depth level × alpha and depth level × theta. (B) *t* values of the regression of alpha and theta upon self-reports at each depth level. Filled triangles denote significant correlations (*P *< 0.05; FDR-corrected). Slopes of the regression fits for the linear mixed model at each individual depth level depicting how (C) alpha and (D) theta amplitudes (in µV) predict experiential self-reports

To evaluate which of the seven NPMs were associated with meditation depth across the five categorical levels, we used a stepwise approach to model reduction for obtaining the significant interactions between NPMs, depth level and group, and between NPMs and depth level when regressed upon self-reported ratings (see Methods). A significant the-way interaction would indicate that slope of the regression of a particular NPM on self-reports was different between depth levels and groups. A significant two-way interaction would indicate that slope of the regression of that NPM on self-reports was different between depth levels (but not across groups). Figure 2A depicts the model-reduction process with columns showing steps and rows showing the three- and two-way interactions modelled at each step. At Step 1, all possible three- and two-way interactions for the seven NPMs were included (depicted in the leftmost column). In subsequent steps, the weakest non-statistically significant (*P *< 0.05; Bonferroni corrected) three-way interactions were dropped (starting from the lowermost row) from the model, ultimately leading to no significant three-way interactions. Then, starting from the weakest two-way interaction all non-significant two-way interactions were dropped. This procedure led to two significant two-way interactions of depth level with alpha (*χ*^2^(4) = 68.23, *P* < 0.001) and theta (*χ*^2^(4) = 55.38, *P* < 0.001) amplitude.

Next, we performed post-hoc *t*-tests to investigate how alpha and theta were related to self-reported ratings at each of the five depth levels (Fig. 2B). The relationship of alpha and theta with depth followed a strikingly complementary pattern. Alpha was significantly negatively correlated with *hindrances* (*t*(81.00) = –4.20, *P *< 0.001 (FDR adjusted)) and positively correlated with *transpersonal qualities* (*t*(87.68)* *= 3.16, *P *= 0.009) and *nonduality* (*t*(75.17)* *= 2.88, *P *= 0.016). Theta was significantly positively correlated with *hindrances* (*t*(66.61)* *= 4.19, *P *< 0.001) and negatively correlated with *transpersonal qualities* (*t*(62.27) =–2.60, *P *= 0.045).

Figure 2C and D shows the estimated slopes of the relationship of self-reported ratings with alpha and theta at different depth levels, while Table 2 provides the statistical values for the pairwise comparisons of the slopes. Both for alpha and theta, the slopes were significantly different between DL1 and all other depth levels, with no significant differences between any other comparisons.

**Table 2.** Pairwise comparisons of the estimated slopes of the relationship between self-reported ratings at different depth levels (DLs) and amplitudes in the alpha and theta bands

DL comparison	Alpha			Theta		
	Estimate	*t*(504)	*P*	Estimate	*t*(504)	*P*
0 > 1	−23.70	−5.82	<0.001	38.29	5.39	<0.001
0 > 2	−22.11	−5.43	<0.001	36.29	5.11	<0.001
0 > 3	−29.02	−7.13	<0.001	46.05	6.49	<0.001
0 > 4	−28.20	−6.93	<0.001	42.93	6.05	<0.001
1 > 2	1.59	0.39	1.00	−2.00	−0.28	0.99
1 > 3	−5.33	−1.31	0.69	7.76	1.09	0.81
1 > 4	−4.50	−1.11	0.80	4.64	0.65	0.97
2 > 3	−6.91	−1.70	0.44	9.76	1.37	0.65
2 > 4	−6.09	−1.50	0.57	6.64	0.93	0.88
3 > 4	0.82	0.20	1.00	−3.12	−0.44	0.99

**Figure 3.**
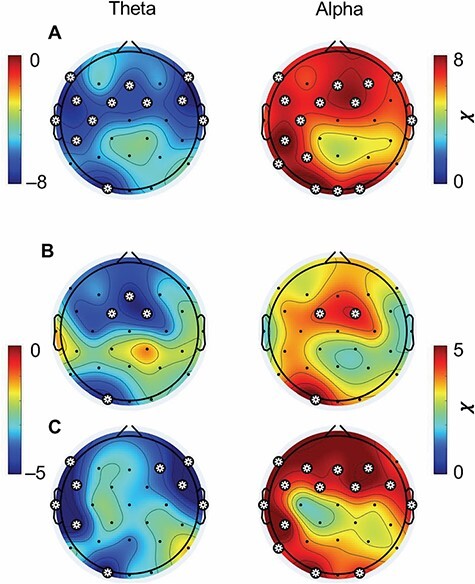
Topographic plots of the relationship between theta (left) and alpha (right) amplitudes and meditation depth in the (A) two groups combined, (B) long-term meditators and (C) controls. Plotted are the square-root of the *χ*^2^ values of the interaction effects between depth level and theta and alpha amplitudes regressed upon self-reports. The values are signed by the direction of relationship, which was negative for theta and positive for alpha at all channels. Asterisks denote channels with significant interaction effects, thresholded for (A) at Bonferroni-corrected *P *< 2.5e–8 and (B–C) at Bonferroni-corrected *P *< 0.025

Figure 3 depicts brain topographies of the strength of the two-way interactions of theta and alpha with depth level. Theta oscillation correlations were observed primarily over medial and lateral prefrontal cortex and occipital cortex (Fig. 3A, left). Alpha oscillation correlations were also observed over similar regions in addition to the left parietal cortex (Fig. 3A, right).

We further tested whether the association between meditation depth and alpha and theta was present in both groups separately (or driven by one group). For this, we modelled interactions of alpha and theta with depth level in the two groups separately. The two interactions were indeed highly significant in both LTMs (alpha: *χ*^2^(4) = 17.30, *P* = 0.002; theta: *χ*^2^(4) = 17.71, *P* = 0.001) and CTLs (alpha: *χ*^2^(4) = 22.04, *P* < 0.001; theta: *χ*^2^(4) = 18.76, *P* < 0.001).

Finally, we investigated if the two groups showed similar or different scalp topographies of the interaction of the two frequency bands with depth level, as the latter may indicate differences in cognitive processes involved in meditation depth or strategies used for evaluating self-reported meditation depth. For the LTMs (Fig. 3B), theta was related negatively to meditation depth over medial frontal and occipital scalp locations, while alpha was related positively to meditation depth over the same locations. For the CTLs (Fig. 3C), theta was negatively related to depth level over left and right lateral frontal and central scalp locations as well as occipital locations. Alpha was related to depth level over medial and bilateral frontal scalp locations along with occipital and left parietal locations.

### Control analyses

While the LTMs engaged in silent self-guided meditation, CTLs engaged in audio-guided meditation. To ensure that differences in topographies between groups (particularly frontal medial and lateral correlations with theta in the LTMs and CTLs, respectively) were not simply due to auditory stimulation, we also analysed the topographies using only the last 6 min of M1 and M2 during which participants were instructed to meditate in silence (Supplementary Fig. S1). These topographies were very similar to the topographies in Fig. 3B–C, indicating that group differences could not be explained by differences in auditory stimulation.

While M1 and M2 practices for CTLs are commonly used as part of mindfulness-based interventions, CH is not. For applicability of our results to mindfulness-based interventions, we also plotted the topographies by excluding the CH block (Supplementary Fig. S2). These topographies were again similar to when the CH block was included.

### Exploratory analysis

#### Meditation depth during chanting

The LTMs reported experiencing deeper meditation states even just through the brief CH intervention before beginning meditation. We explored if this deepening was accompanied by brain mechanisms similar to meditation, specifically by a reduction in frontal midline theta. Supplementary Figure S3 shows the theta topography for the LTMs of CH compared to BL, which again showed a locus at midline frontal regions although at a somewhat lenient threshold (*P *< 0.025; uncorrected).

#### Meditation depth and physiology

**Figure 4.**
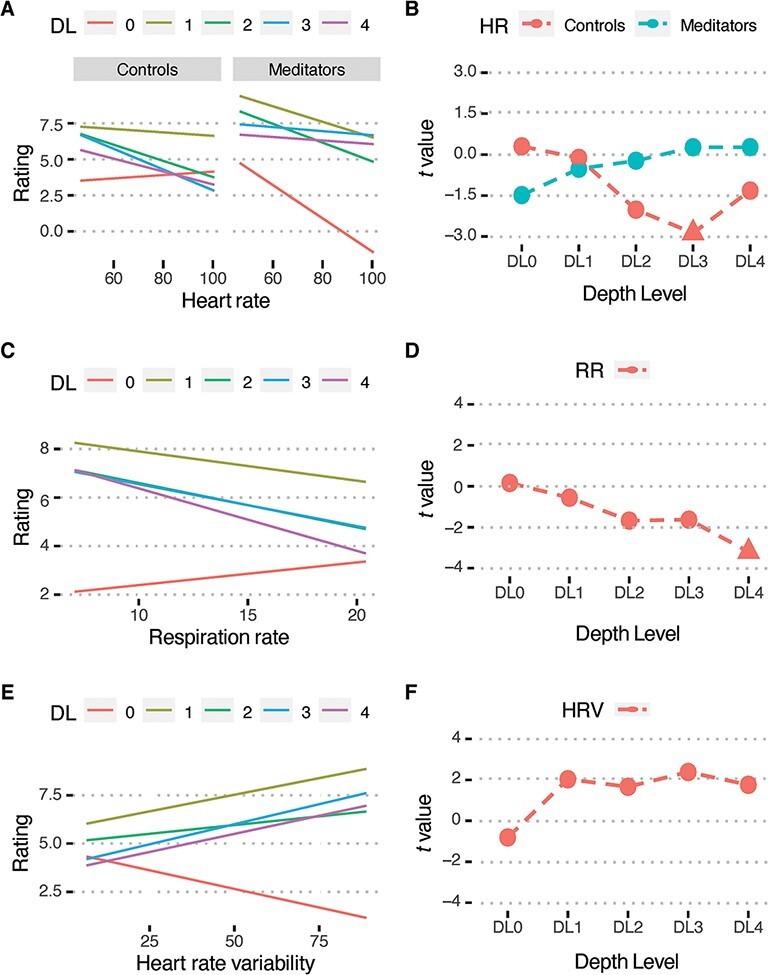
Slopes of regression fits for individual depth levels depicting how self-reported ratings are predicted by (A) heart rate (in cycles/minute) for the two groups, controls and long-term meditators and (C) respiration rate (in cycles/minute) and (E) heart-rate variability across groups. *t* values of the correlation between subjective reports of depth at each of the five depth levels with (B) heart rate for the two groups, (D) respiration rate and (F) heart-rate variability. Filled triangles denote significant correlations (*P *< 0.05; FDR-corrected) at that depth level

Our planned analysis did not reveal a significant relationship between meditation depth and the three physiological measures (HR, RR, HRV). It is however possible that if there were such a relationship, it was being explained away by the neural correlations. We performed exploratory analysis by regressing the three- and two-way interactions from only RR, HR and HRV upon self-reports. Model reduction revealed three significant interactions. There was a three-way interaction of HR with *depthlevel* and *group* (*χ*^2^(4) = 13.48, *P* = 0.009). Post-hoc comparisons revealed that this was because the change in slope across depth levels was different for the two groups (Fig. 4A). While the slope increased for the LTMs, it decreased for the CTLs. When regressing HR on individual depth levels, we found a significant negative relationship between HR and DL3 for the CTLs (Fig. 4B; *t*(20.20) = −2.89, *P *= 0.045), but no relationship between HR and any of the individual depth levels was observed in LTMs. There were also significant two-way interactions of RR (*χ*^2^(4) = 14.79, *P* = 0.005) and HRV (*χ*^2^(4) = 13.48, *P* = 0.009) with depth level. For RR, the slope of its relationships with self-reports decreased with depth level, i.e. at greater depth levels there was a more negative relationship between self-reports and RR (Fig. 4C). Pairwise comparison revealed that the slope of RR at DL0 was significantly greater than for DL4 (*t*(354) = 3.18, *P* = 0.014). At individual depth levels, RR was significantly (negatively) correlated to DL4 (Fig. 4D; *t*(86.00) =−3.42, *P *= 0.005). The slopes of the relationship of HRV increased with depth level, i.e. at greater depth levels there was a more positive relationship between self-reports and HRV (Fig. 4E). Pairwise comparisons revealed that slopes of HRV for DL0 were significantly smaller compared to DL1 (*t*(354) = −3.41, *P* = 0.006), DL3 (*t*(354) = −3.74, *P* = 0.002) and DL4 (*t*(354) = −3.56, *P* = 0.004). None of the correlations of HRV with individual depth levels survived FDR correction (Fig. 4F).

#### Neural correlates of individual depth levels

For our planned analysis, we used a statistical approach that reveals the neural correlates that differentiate the different depth levels. Such an analysis approach however would miss neural correlates that are specific to a particular depth level (and do not change reliably across depth levels). Hence, as an exploratory analysis, we regressed the four neural measures (theta, alpha, beta and gamma) and their interaction with *group* upon each of the five depth levels individually (Supplementary Fig. S4). As above, we found that DL0 was significantly positively associated with theta (*χ*^2^(1) = 17.52, *P* < 0.001; *t*(66) = 4.10; *P* < 0.001) and negatively associated with alpha (*χ*^2^(1) = 17.68, *P* < 0.001; *t*(80.5) = −4.10; *P* < 0.001), with no interaction of either with group. DL1 and DL2 were not associated with any of the frequency bands. Again, as above, DL3 was significantly negatively associated with theta (*χ*^2^(1) = 5.92, *P* = 0.015; *t*(77.9) = −2.38; *P* = 0.020) and negatively associated with alpha (*χ*^2^(1) = 14.65, *P* < 0.001; *t*(71.4) = 3.74; *P* < 0.001), with no interaction of either with group. DL3 was also significantly negatively associated with beta (*χ*^2^(1) = 4.74, *P* = 0.029; *t*(44.2) =–2.13; *P* = 0.039) and positively associated with gamma (*χ*^2^(1) = 7.96, *P* = 0.005; *t*(67) =–2.77; *P* = 0.007) with neither interacting with group. For DL4, we observed an interaction of the theta amplitude with group (*χ*^2^(1) = 4.06, *P* = 0.044). This interaction was due to a negative association between theta and DL4 only in the LTMs (*t*(57.5) = −2.44; *P* = 0.018; Supplementary Fig. S4, DL4 theta, grey line) and not CTLs (*t*(42) = −0.17; *P* = 0.87; black line). Finally, alpha was positively associated with DL4 (*χ*^2^(4) = 7.52, *P* = 0.006; *t*(42) = 2.67; *P* = 0.009).

## Discussion

The goal of this study was to examine the relationship of the neural and physiological correlates of self-reported meditation depth in LTMs of the same contemplative tradition versus demographically matched meditation-naïve adults. We expected that, compared to CTLs, LTMs would experience a greater meditation depth (Hypothesis 1), and that across participants a greater meditation depth would be positively associated with alpha, theta, gamma and HRV and negatively associated with beta, RR and HR (Hypothesis 2).

We found evidence supporting Hypothesis 1. Compared to LTMs, the CTLs reported overall significantly more *hindrances* across all blocks (BL, M1 and M2). Both groups reported higher *relaxation* and *concentration* during meditation compared to BL. The deepest experiences—*transpersonal qualities* and *nonduality*—were significantly heightened in the LTMs following M1 and M2 compared to BL but not in the CTLs. Because the two groups performed different meditation techniques, these differences may be due to either the difference in the amount of meditation training between groups (several decades vs none), or a difference in meditation practices themselves (for example, LTMs meditation techniques involved an explicit component of meditative absorption, while the CTLs engaged in guided focused attention on breath and then loving-kindness).

The self-reported meditation depth (MEDEQ) results suggest that even meditation-naïve individuals can experience relaxation and concentration following the commonly known mindfulness of breath and loving-kindness meditation practices. They also indicate that LTMs experience fewer hindrances such as distraction and drowsiness not only during meditation, but also when performing a mundane task like listening to a podcast at BL. Moreover, while it was expected that LTMs (and not CTLs) would experience the deepest levels of meditation experiences following meditation practices M1 and M2, we found that LTMs reported experiencing these deepest levels also when listening to CH at a level comparable to M1 and M2. This suggests that regular training could enable deep meditation experiences even through the practice of CH. Our results indicate a need for better understanding CH practices in the future. Interestingly, we did not observe trait differences during BL for any of the five depth levels. The lack of trait differences in the MEDEQ may stem from the fact that it is a questionnaire that measures state modulations, and when providing self-reports about experience, the absolute value that an individual provides as a Likert rating would be expected to be normalized to their own (and not everyone’s) experience. Another reason for the lack of trait differences during BL may be that participants were actively engaged in the task of listening to a podcast. Previously, trait differences between LTMs and novices have been mostly reported based on a passive resting BL (Cahn and Polich 2006). We did not use a resting BL because we thought that during such a condition the meditators may habitually enter into a state of meditation, thus making it difficult to modulate meditation depth across blocks. We recommend future studies to use both an active and resting BL for measuring both state and trait effects.

Hypothesis 2 was that meditation depth would be associated with one or more of the seven NPMs, as determined by the change in the slope of the correlation between an NPM and self-reported rating across the five depth levels. We found that meditation depth was strongly associated with alpha and theta amplitudes, respectively. While the relationship of meditation depth with alpha was in the expected positive direction, its relationship with theta was opposite to the expected direction. In fact, the two neural measures were correlated to self-reported ratings across the five depth levels in a precisely opposite manner. Theta correlated positively with *hindrances*, whereas alpha correlated negatively. At progressively deeper levels, however, the self-reports became more positively correlated with alpha and more negatively correlated with theta.

Hypothesis 2a was that the association between meditation depth and NPMs would be different between LTMs vs CTLs. We did not find this to be the case. In fact, a similar association of alpha and theta with meditation depth was strongly significant within each group. This suggests that, despite the two groups engaging in different styles of meditation practices and having vastly different amounts of meditation experience, change in self-reported meditation depth relies on somewhat similar aspects of brain function. The lack of group difference in our findings is also consistent with past studies that have found changes in alpha and theta during meditation irrespective of the amount of meditation training or the meditation tradition (Cahn and Polich 2006; Lomas *et al.* 2015) and shown similar neural correlates of effortless concentration between LTMs and novices (van Lutterveld *et al.* 2017). Another reason we may not have observed group differences was because of the way we conducted our planned analysis, namely, by associating NPMs with self-reports across all five depth levels. Such an analysis disallowed measurement of group differences at the deepest levels separately, which is where there may have been a difference between groups. Indeed, post-hoc analysis revealed that the deepest meditation experiences (*nonduality*) were negatively related with theta only in the LTMs and not in CTLs.

Alpha oscillations are closely linked to inhibitory processing and are often related to suppression of distractors during attentional processing (Jensen and Mazaheri 2010; Klimesch 2012; Clayton *et al.* 2015), whereas theta oscillations (particularly frontal theta, as we observed) are generally related to attentional monitoring, control or selection (Clayton *et al.* 2015). As meditation deepens, we would expect greater suppression of distractors and thus increased alpha. Moreover, once the distractors have been suppressed during a deep state, there would be less need for cognitive control due to a more effortless quality of concentration, which may correspond to reduction in theta. In this sense, alpha and theta might act in a complementary way with deepening meditation. This complementary relationship was also evident in the similar set of brain regions where alpha and theta were related to meditation depth—lateral and medial prefrontal and occipital cortex.

While the association of depth with alpha and theta was present over the occipital channels for both groups—indicating reduced visual sensory processing—there were group differences in where these associations were centred over the frontal channels. Notably, the association of greater meditation depth and lesser theta was present over medial-frontal scalp locations in LTMs and lateral-frontal scalp locations in CTLs. Medial-frontal theta is a classical signature of conflict monitoring and inhibitory control signals (Cohen 2014; Clayton *et al.* 2015). During meditation practice, such a cognitive process would be required, for example, to monitor the contents of consciousness, and detect conflict if actual conscious content (in the form of a distractor) does not match what one is expecting to meditate on. Moreover, growing evidence suggests that lateral-frontal theta is related to attentional regulation (Fellrath *et al.* 2016; Rajan *et al.* 2019; Spooner *et al.* 2020). From a cognitive standpoint, conflict monitoring provides control signals for attention regulation (i.e. more conflict triggers the need for more regulation) (Botvinick *et al.* 2001; Kerns *et al.* 2004), which again would be required during meditation practice to upregulate the target and downregulate distractors. It is thus possible that the different scalp locations of negative correlations of meditation depth with theta oscillations were related to different aspects of executive processes downregulated during deeper meditation. This would in turn imply that trained meditators were able to downregulate executive processing at an earlier stage of processing. While interesting from a mechanistic viewpoint, this interpretation remains speculative in the absence of neural source localization, and behavioural data. Moreover, because the two groups were performing different practices during M1 and M2, it is unclear if the differences in theta correlations were due to differences in the amount of training (many years versus none) or the type of meditation techniques (deep absorption versus mindfulness of breath/loving kindness). In other words, the differences in topographies of theta correlation in the LTMs vs. CTLs may have been due to the absorptive nature of practices that LTMs engaged in. Although similar reduction in medial-frontal theta during a response inhibition task in LTMs trained in practices (Vipassana) similar to our control group suggests that our reduced medial-frontal theta may be a general feature of prolonged meditation training (Andreu *et al.* 2019).

Although meditation depth was not related to psychophysiological measures when modelled in combination with the neural measures, we did observe a relationship when physiology was modelled separately. This indicates that neural activity in alpha and theta bands effectively ‘explained away’ variance of the physiological measures. RR was negatively correlated with the deepest (*nondual*) meditation experiences. This extends previous anecdotal evidence of slowing down of RR accompanying deep meditation experiences (Corby *et al.* 1978). In the LTMs, moreover the baseline RR was negatively correlated with the amount of meditation training (Supplementary Results), replicating a recent study (Wielgosz *et al.* 2016). We also observed that HR was negatively correlated with *transpersonal qualities* in CTLs but not LTMs. While unexpected, this is an interesting observation with respect to theories that propose subjective experience relies on predictive neural processing about interoceptive signals (Gallagher 2005; Seth 2013). In this sense, HR signals may have constituted the evaluation of experience of *transpersonal qualities* in the CTLs but not LTMs, indicating that long-term meditation training may reduce the influence of certain interoceptive signals as somatic ‘markers’ (Damasio 1999) of subjective experience.

Finally, meditation depth was not related to beta and gamma oscillations, previously associated with different subjective experiences during meditation (Dor-Ziderman *et al.* 2016; van Lutterveld *et al.* 2017). We suspect two reasons for this. First, we used multivariate statistical models unlike previous studies that have correlated different frequency bands with self-reports using separate statistical tests for each band. The multivariate approach is advantageous because it enables a parsimonious discovery of correlates while accounting for covariation between them. Second, we measured the substrates of a relatively broad concept of meditation depth with five levels encompassing a larger variety of experiences including the previously studied ones. This enabled us to discover more general processes involved in deeper meditation compared to the specific processes investigated previously. Indeed, exploratory analysis at individual depth levels, which correspond to more specific experiences, revealed that beta and gamma amplitudes were negatively and positively related to *transpersonal qualities*, respectively. The beta band negative correlation parallels the previously observed negative relationship between beta band activity and self-transcending experiences (Dor-Ziderman *et al.* 2013, 2016). The gamma band positive correlation however was in the opposite direction to the previously observed negative correlation between gamma band EEG activity and effortless concentration (van Lutterveld *et al.* 2017). There is growing evidence for the role of gamma-band activity in both trait- and state-based differences with meditation training (Lutz *et al.* 2004; Braboszcz *et al.* 2017; Katyal and Goldin 2021). Functionally, gamma band activity has been related to conscious binding and attention (Engel and Singer 2001; Jensen *et al.* 2007), which may have been involved in increased *transpersonal qualities*.

While the two groups performed different meditation practices (M1 and M2), the baseline and chanting blocks were similar for them. When comparing these two blocks between the two groups (Supplementary Results), we found that only the long-term meditators had an increased gamma-band activity and increased heart-rate during chanting compared to baseline, possibly indicating greater attention and arousal during chanting. Interestingly, despite the increased heart-rate, there was a trend across both groups for decrease in respiration rate from BL to chanting, suggesting that the chanting practice in long-term meditators may involve a simultaneous activation of the sympathetic and parasympathetic system.

We administered the MEDEQ to assess participants’ meditation experience after relatively long meditation blocks (15–20 min). In contrast, some previous studies have used experience sampling (Brandmeyer and Delorme 2018) or online experience reporting (van Lutterveld *et al.* 2017) methods. We used longer meditation blocks to allow participants sufficient time to enter deeper levels of meditation without being distracted by the need to introspect and report their experience. However, because accurate recollection of experience is known to be difficult, our long blocks may have come at the cost of reliability of recollection. To inform future studies about the meditation duration vs. depth trade off, we examined the alpha and theta amplitude’s correlation with meditation depth as a function of duration from the beginning of the block over which we averaged the amplitude. We found that in LTMs, significant correlation for both alpha and theta emerged around 8 min and plateaued around 12 min from the beginning of blocks M1 and M2. Surprisingly, for the CTLs, the correlation between meditation depth and alpha and theta emerged even as early as 2 min into the meditation blocks. One possible reason for this difference in the emergence of the correlation between groups could have been trait differences in deep experiences between groups. For example, LTMs may have already started off at a deeper experiential level at baseline, thus prolonging the difference in experiential depth to emerge during meditation. CTLs, on the other hand, did not experience any depth at BL and thus entered states with fewer *hindrances* and more *relaxation* soon after meditation began. Such an explanation would be supported by the trend for fewer *hindrances* in the LTMs compared to CTLs during baseline. Another potential reason is that the experience of the LTMs was progressing over the meditation period, while the CTLs did not experience much change in experience from the beginning.

One limitation of the present study is that the two groups engaged in different meditation techniques. While the LTMs engaged in advanced absorptive practices, we used more widely known mindfulness of breath and loving-kindness meditation practices for the CTLs. As a result, the differences between groups in our results could also be attributed to the differences in the meditation techniques. One way this concern could be addressed in future studies is by using guided absorptive meditation practices that are common for LTMs and novices, which are sufficiently close to the meditation techniques practiced by the LTMs. Future studies could also have both groups doing both absorptive and mindfulness/loving-kindness practices, as a better way to compare the different styles. A second limitation is that by measuring neural correlations of the change in self-reports within a participant, we were unable to measure the neural correlates of the absolute level of meditation depth. Measuring an absolute correlation of an experiential measure is challenging because individuals can only report their experiences relative to themselves. This could however be circumvented in future studies by using second person techniques, where an interviewer uses phenomenological techniques to assess and rate a participant’s experience, which is then correlated with third-person (physiological) measures. Another limitation is that we did not measure high-density EEGs and were thus unable to source localize the lateral prefrontal regions where theta was related to meditation depth.

To summarize, previous meta-analyses of the neurophysiology literature on meditation training have found alpha and theta oscillations to be the most reliable neurophysiological signatures (Cahn and Polich 2006; Lomas *et al.* 2015). However, until now, no studies to our knowledge have empirically differentiated the distinct roles played by these two frequency bands in meditation. This is because previous studies did not take different levels of meditation depth into account. By doing so, our results strongly indicate that while alpha increases as meditation experiences deepen, theta decreases. Topographies of the association of theta with meditation depth combined with their known functional cognitive roles moreover indicate that meditation depth depends on reduced executive processing, at the level of monitoring in the LTMs and at the level of control in the CTLs. Finally, as our meditation-naïve control group engaged in mindfulness practices used in mindfulness-based interventions for improving mental health and well-being in clinical and positive psychology settings, our results are applicable to such scenarios.

## Supplementary Material

niab042_Supp

## Data Availability

Study material, postprocessed data and the R code for statistical analysis and generating figures are available at osf.io/sfkte/. MATLAB code for the entire pre-processing pipeline is available at github.com/sucharitk/meddepth. Full individual participant data set will be made available upon request.
