# Supplementary material for: Alpha and theta oscillations are inversely related to progressive levels of meditation depth
Source: Neurosci Conscious. 2021 Nov 29;2021(1):niab042. doi: 10.1093/nc/niab042 (PMC8633885; doi:10.1093/nc/niab042)
Supplement: niab042_Supp [file niab042_supp.zip › medbrainheart-nconsc-supp.pdf]

Supplementary Materials

Supplementary Methods

Participant recruitment

Sample-size justification

Our goal was to recruit highly-trained meditators with several decades' long meditation experience so that they were able to enter the deepest meditation experiences. Past studies investigating neural correlates of subjective experience during meditation have used sample sizes of expert meditators ranging from n = 11 (Dor-Ziderman et al., 2016) or 12 (Brandmeyer & Delorme, 2016; Dor-Ziderman et al., 2013) to n = 16 (van Lutterveld et al., 2017) reflecting the challenge associated with recruiting highly trained meditators. Of these studies, the one with the larger sample size recruited meditators with only an intermediate level of training (>5 years) as they investigated meditation experiences akin to an intermediate depth level (DL2). In order to measure experiences corresponding to DL3 and DL4, we expected to use more advanced meditators. We therefore aimed to recruit 12 or more long-term meditators in our study consistent with past work (Brandmeyer & Delorme, 2016; Dor-Ziderman et al., 2013, 2016).

Long-term meditators and their meditation tradition

LTMs were recruited from a single *Ananda Marga* meditation tradition through their regional and national email lists.

Ananda Marga (English translation: Path of Bliss; anandamarga.org) is a tradition of Tantra Yoga meditation originating in India and founded by Prabhat Rainjan Sarkar (also

known as Shrii Shrii Anandamurti). While Tantra Yoga meditation is believed to have been practiced in esoteric settings for several thousand years (Harper & Brown, 2002), Sarkar systematised these practices within a rational and pragmatic philosophical framework (Anandamurti, 1998; Taraka & Acyutananda Avadhuta, 2014) and made them universally available. An *Ananda Marga* practitioner is taught a fully codified sequence of six progressive meditation lessons by an ordained teacher (an *acharya*) over the span of several years. The teacher imparts each new meditation lesson to the meditator after ascertaining their progress on previous lessons. The six lessons themselves are part of the classical eight limbs of Yoga as codified by Patainjali in the Yoga Sutras (Anandamurti, 2010; Bharati, 2001). The first and sixth lessons are considered the main meditation lessons, and are the ones we asked the LTMs to practice in the present study (see the Meditation Blocks section below), while the four remaining are considered "helper" lessons. The first lesson involves concentrating with eyes closed on an internally visualised point of light while silently repeating a personal *mantra* (assigned by the instructor when first learning this practice), where the meaning of the *mantra* embodies the idea that one is dissolving their egocentrically-based feeling of self-consciousness (e.g., "I am someone") into a transpersonal feeling of consciousness ("I am everything"). The sixth lesson is a "non-dual" meditation practice, which involves contemplating suspension of one's intentional agency (e.g., "I am meditating", or x is doing y) into a "transcendental consciousness" such that there remains a feeling of neither y nor x. The idea of transcendental consciousness in *Ananda Marga* philosophy (Anandamurti, 1998) has parallels to a Husserlian transcendental consciousness in that it evades any reflection at all (Hewitson, 2014) in contrast to the more widely-known idea of "pure consciousness" described in "Transcendental Meditation" literature, which has been previously investigated

using introspection (Travis & Pearson, 2000). Besides the first and sixth lessons, the other lessons of meditation are supposed to help the meditator cultivate better single-pointed sustained focus on the object of meditation (*dharana*), allow control over one's breathing and as a result the parasympathetic nervous system (*pranayama*), and provide visualisation tools for withdrawing attention from external senses to enable better concentration on the meditated object (*pratyahara*). Besides these six lessons, Ananda Marga meditators are also encouraged to follow moral guidelines codified in the Yogic system of *yama* and *niyama*, as well as practice yoga postures (*asanas*) to cultivate a healthy body and mind (Taraka & Acyutananda Avadhuta, 2014). The overall goal of *Ananda Marga* meditation practices as stated in their motto is twofold: 1) at a personal level, to attain deeper levels of meditative trance (*samadhi*), which in turn lead to a gradually better intuition about the nature of one's consciousness, and 2) to dedicate one's life work for social and ecological welfare.

One reason for choosing this meditation tradition was that an older study from this tradition reported a practitioner entering a deep state of meditative trance (*samadhi*) during the study, but no follow-up investigation on this type of meditation practice has been conducted since (Corby et al., 1978). A second reason was that because all meditation practitioners did similar and well-specified practices in the same sequence, we can make stronger inferences regarding what the neural correlates in the LTMs may reflect. LTMs were included based on >10 years of regular daily meditation practice. Eight of the thirteen LTMs were ordained monastics. LTMs reported engaging in multiple practices as part of their daily regimen, which included 2—3 hours of daily sitting meditation.

Control participants

CTLs were recruited through online forums and by posting fliers in public and university libraries in the Central California area and were required to be meditation-naïve.

Missing data

Due to technical difficulties, we did not obtain pulse and respiration measures for one of the CTLs. On another CTL, measurement of pulse could not be distinguished from noise, so was excluded. On one LTM, pulse measurement was excluded for the M2 block where it was indistinguishable from noise. For the MEDEQ, only 3 (out of 112 total possible post-block assessments) were missing across all participants.

Meditation blocks

For BL, participants kept their eyes closed and listened to a history-based podcast (*The Memory Palace* Episode 36) narrating a brief biography of the child prodigy William J. Sidis (thememorypalace.us/six-scenes-in-the-life-of-william-james-sidis-wonderful-boy). For CH, with eyes closed participants listened to a musical chant (available online at: osf.io/sfkte) involving a repetitive singing of a sanskrit mantra, *Baba Nam Kevalam*. LTMs were familiar with this chant and could use this as a tool for meditation according to its meaning ("love is the essence of everything"). CTLs were also told the meaning of the chant and asked to simply listen to the music.

For M1 and M2, the LTMs engaged in two different self-guided meditation practices that are part of their daily regimen (first and sixth lessons of *Ananda Marga* meditation; see above). As these involve traditional elements like personalised *mantra* based on a process of formal initiation into *Ananda Marga* meditation practices, and esoteric concepts like merging one's consciousness into an extended transpersonal consciousness, we could not use the same practices with CTLs. Instead, we used more commonly known meditation practices for

the CTLs. For M1 in CTLs, we chose a focused attention on breath mindfulness meditation practice (by Tara Brach from tarabrach.com). We chose this practice because we thought it would overlap with M1 in LTMs in cognitive processing terms at least partly because of the instruction to try to retain focused attention on a mental object. A second reason for choosing this practice was that we expected to manipulate DL1 (relaxation) and DL2 (concentration) ratings in the CTLs through this practice and thus be able to measure their neurophysiological correlates in the CTLs. We did not expect that the CTLs could be instructed into the kind of non-dual absorption that LTMs engaged for M2. Instead, for M2 in CTLs we chose the meditation practice of loving-kindness towards a stranger, a close other, and oneself (by Elisha Goldstein from elishagoldstein.com), which we expected would manipulate DL3 (transpersonal qualities) in the CTLs. Meditation instructions for these audio-guided meditation practices in CTLs were delivered through headphones.

Because the MEDEQ measures subjective experiences independent of the type of meditation practiced, it allows us to combine or compare the two groups despite differences in M1 and M2.

EEG and physiology preprocessing

We used EEGLAB (Delorme & Makeig, 2004) and customized MATLAB code to conduct EEG data analysis. Raw EEG data were first downsampled to 250 Hz then band-pass filtered between 0.1–80 Hz, notch filtered with a 1 Hz bandwidth to exclude electrical line noise at 60 Hz, and re-referenced to the average of all electrodes. We then used Independent Component Analysis (ICA; as implemented in EEGLAB) to remove ocular and muscular artifacts (Delorme et al., 2007). Despite ICA, however, the four peripheral-most lateral channels (FT9, FT10,

TP9, TP10) still appeared to be contaminated with muscle artifacts in many subjects. Moreover, midway through the study, the lead of one of the front-polar channels (FP2) was corrupted. Accordingly, for the final analysis we excluded a total of six channels (FT9, FT10, TP9, TP10, FP1, FP2). Results were however very similar when these channels were included in analysis.

To compute the seven neuro-physiological measures, we first split each of the four measurement periods, BL, CH, M1 and M2 into multiple 60-sec epochs with a 40-sec overlap between adjacent epochs. Overlapping windows were used to ensure that we did not miss any cycles of the physiological variables, which modulate at relatively low frequencies, at the window edges. As M1 was 20 minutes and M2 was 15 minutes, we used only the last 15 minutes of M1 to match the duration of M2. We used the entire duration of BL and CH for analysis. We then calculated the average amplitude in four frequency bands corresponding to *theta* (4–7 Hz), *alpha* (7–13 Hz), *beta* (15–25 Hz), *gamma* (30–50 Hz) oscillations at each channel using MATLAB’s FFT function and then averaged these measures across all epochs and all channels per block. Respiration-rate (RR), heart-rate (HR) and heart-rate variability (HRV) were also calculated in similar 60-sec epochs. For calculating RR, we filtered the respiration data between 0.04—0.4 Hz and calculated the median of the inverse time difference between peaks corresponding to the inhale phase within each epoch. Epochs where peak-duration differences were highly variable (interquartile range > 5) were marked as artifactual and excluded from analysis; results were similar for a wide range of interquartile range thresholds (e.g., 5 < interquartile range < 10). Heart measures, HR and HRV, were calculated in the 60-epochs using *HRVTool* toolbox (version 0.3) for MATLAB (Vollmer, 2019), which provides a function to filter out artifactual pulses. HRV was calculated

in the high-frequency range (0.15–0.4 Hz) consistent with previous literature (e.g., Lumma et al., 2015).

Statistical analyses

Association between amount of meditation training and neurophysiological measures

We measured the amount of meditation training in years of training. To test the relationship between the years of meditation training and NPMs during BL listening to podcast block, we used a linear mixed model where we regressed each of the NPMs across the multiple 60-sec epochs of the BL block on the duration of training with a random intercept for each participant. Post-hoc analysis was conducted using the *emtrends* function of the *emmeans* package, which calculates the degrees of freedom using the Kenword-Roger method.

MEDEQ questions used in our study

1. I experienced equanimity and inner peace (DL 2)
2. My mind was constantly distracted by thoughts (DL 0)
3. The difference between subject and object disappeared (DL 4)
4. I gradually became more calm and patient (DL 1)
5. I could observe my thoughts from a distance (DL 2)
6. My mind was alert and clear (DL 2)
7. I felt sleepy and was dozing off (DL 0)
8. I felt love, surrender, connection (DL 3)
9. The feeling of time disappeared (DL 3)
10. I experienced a sense of boundless joy (DL 3)
11. My awareness expanded to an infinite space (DL 4)
12. I felt being accepted unconditionally (DL 3)
13. My mind was empty of thoughts, emotions and sensations (DL 4)
14. I experienced humility, grace, gratitude (DL 3)

Supplementary Figures

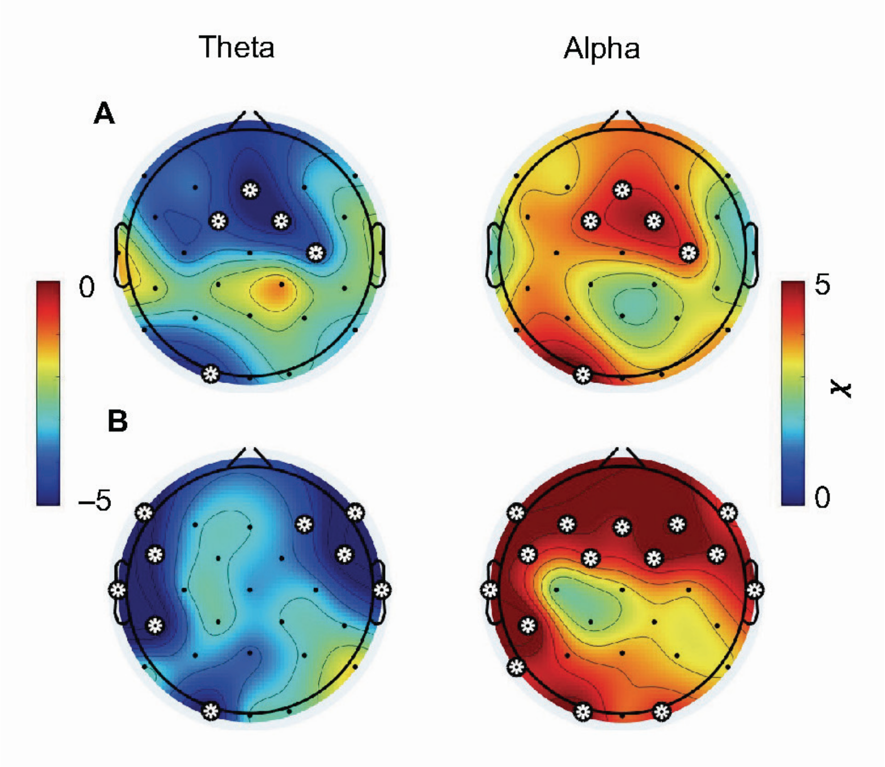

Supplementary Figure 1. Topographic plots of the relationship between theta (left) and alpha (right) amplitudes and meditation depth in **A)** long-term meditators, and **B)** controls for only the last 6 minutes of the meditation block. Plotted are the square-root of the χ^2 values of the interaction effects between depth-level, and theta and alpha amplitude regressed upon self-reports. The values are signed by the direction of relationship, which was negative for theta and positive for alpha at all channels. Channels with asterisks show significant interaction effects thresholded at Bonferroni-corrected $p < 0.025$.

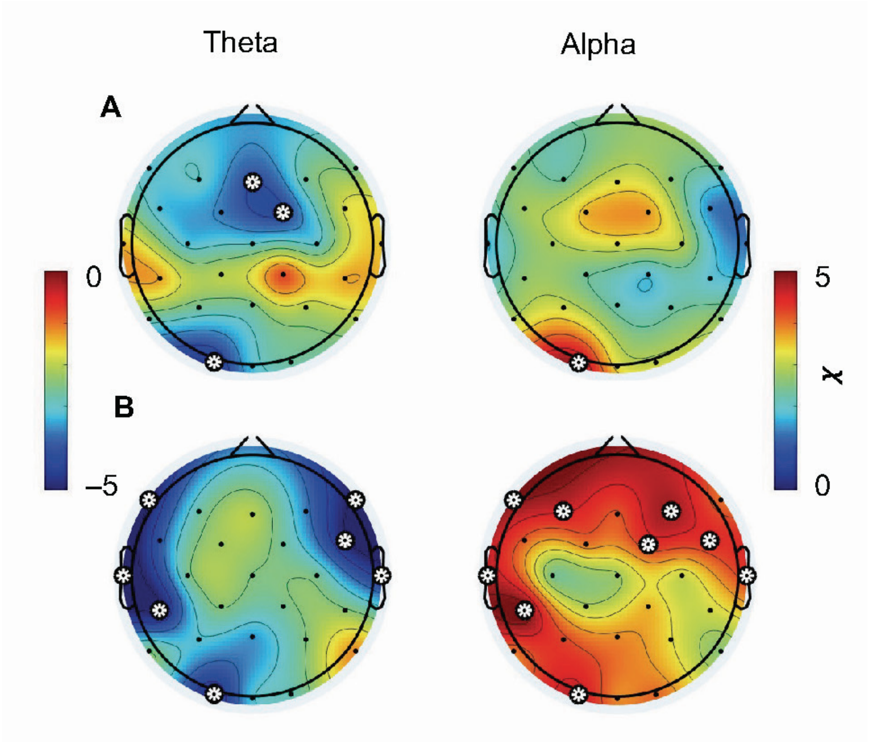

Supplementary Figure 2. Topographic plots of the relationship between theta (left) and alpha (right) amplitudes and meditation depth in **A)** long-term meditators, and **B)** controls evaluated while excluding the chanting block. Channels with asterisks show significant interaction effects thresholded at Bonferroni-corrected $p < 0.025$.

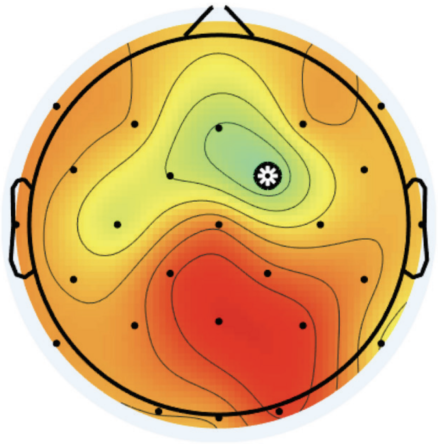

Supplementary Figure 3. Topographic plots of the relationship between theta amplitude and meditation depth in long-term meditators for only the chanting block. Channels with asterisks show significant interaction effects thresholded at $p < 0.025$ (uncorrected).

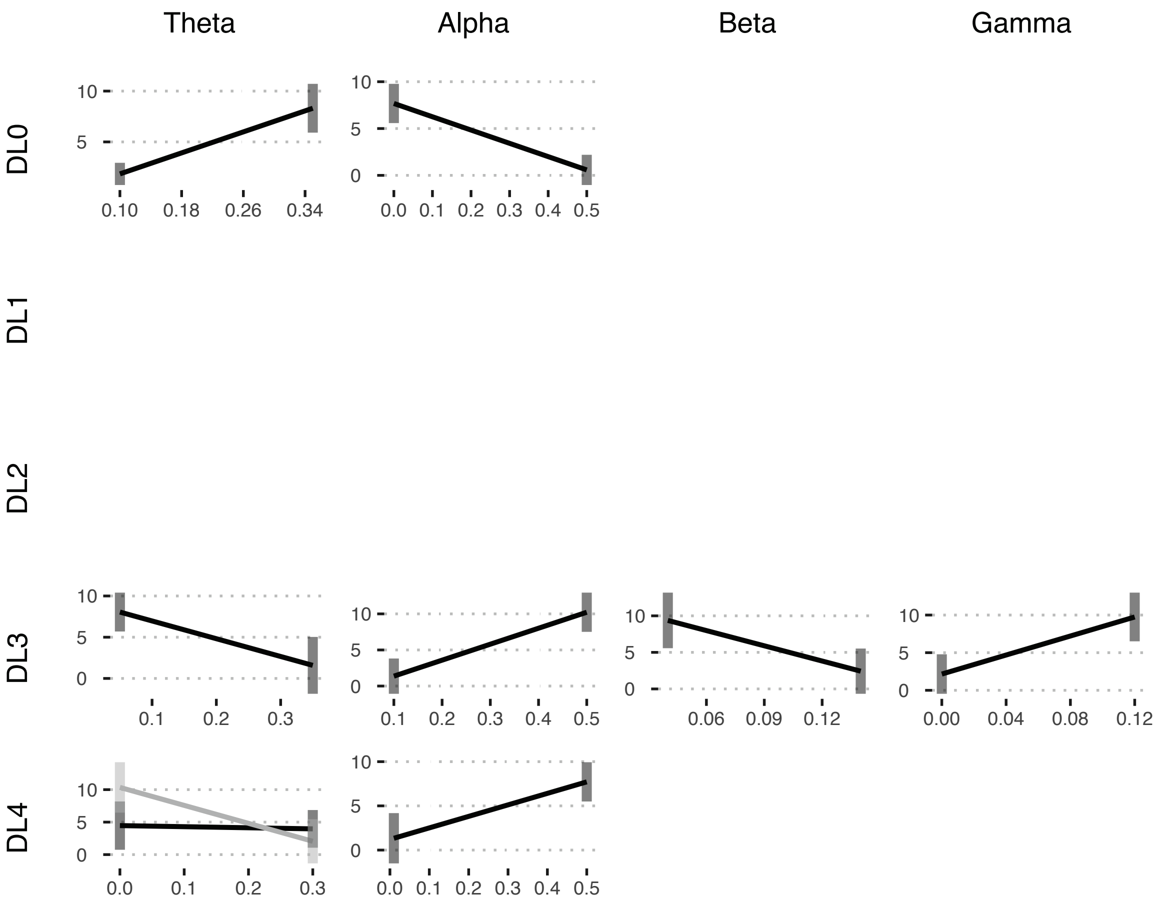

Supplementary Figure 4. Linear mixed model regression estimates of the relationship between the four EEG frequency bands (theta, alpha, beta and gamma; depicted along the columns) and self-reported rating at the five depth levels (DL0–4; depicted along the rows). Error bars at the extreme values show the 95% confidence intervals. Only panels with either a significant interaction ($p < 0.05$) between the frequency band and group, or a significant main effect ($p < 0.05$) of the frequency band upon the self-reported ratings are shown. The interaction with group was significant only for theta at DL4 where the black line is for the CTLs and the grey line for the LTMs. The remain panels only had a main effect of the frequency band.

Results

Neural and physiological changes with amount of meditation training

We examined whether the duration of meditation training (in years) in the LTMs predicted any of the NPMs during BL. To do so, we regressed the number of years of practice upon the seven NPMs.

The amount of meditation training was not related to any of the four frequency bands (theta: $\chi^2(1) = 0.58$, $p = 0.45$; alpha: $\chi^2(1) = 0.02$, $p = 0.88$; beta: $\chi^2(1) \sim 0$, $p = 0.95$; gamma: $\chi^2(1) = 2.75$, $p = 0.10$) or heart-rate ($\chi^2(1) = 0.12$, $p = 0.73$). There was however a significant negative correlation between the amount of training and respiration rate ($\chi^2(1) = 7.61$, $p = 0.006$; $t(9.93) = -2.76$, $p = 0.020$; Supplementary Figure 5A).

We also evaluated the relationship between the amount of meditation training in the LTMs and the change in

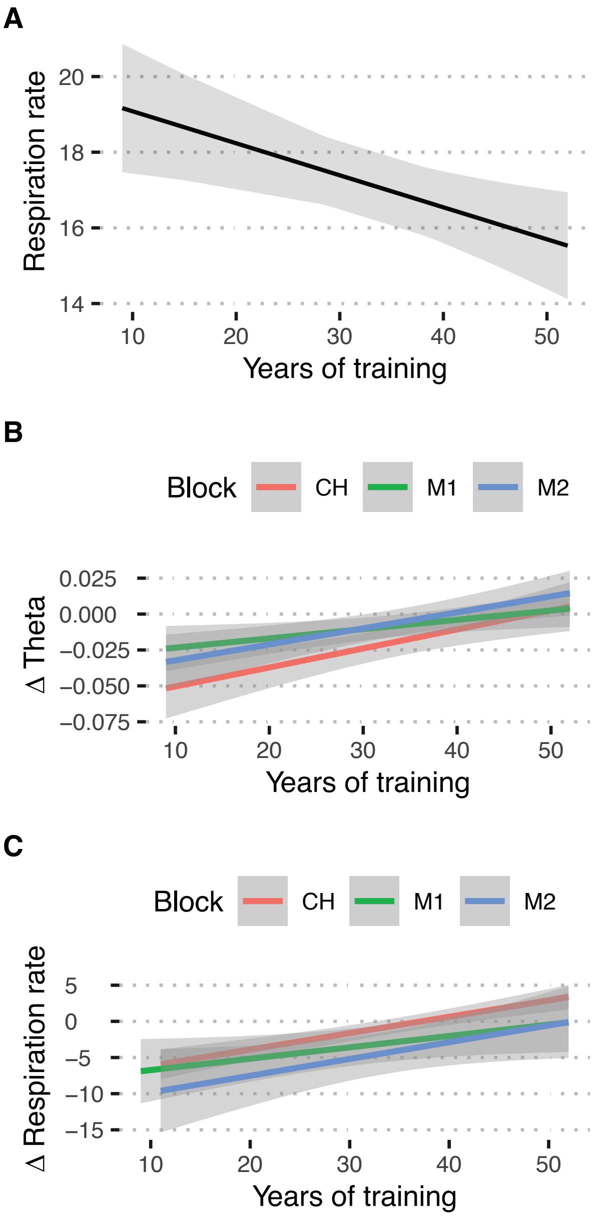

Supplementary Figure 5. Linear mixed model regression estimates of the relationship between the amount of meditation training (in years) in the LTMs and A) respiration rate (cycles/min) showing a significant (p = 0.020) negative relationship between the two, B) change in theta amplitude (in µV) from BL to CH, M1 and M2 showing a significant (p = 0.007) positive relationship, and C) change in respiration rate from BL to CH, M1 and M2 showing a significant (p = 0.040) negative positive relationship.

NPMs from BL to CH, M1, and M2. Such an analysis gives an idea of how meditation training may change the ability to manipulate the NPMs towards a particular experience. For this analysis, we regressed the amount of meditation training upon each of the NPMs over CH, M1 and M2 after subtracting BL. We found a significant positive relationship between the amount of meditation training and change in theta ($\chi^2(1) = 10.90$, $p = 0.001$; $t(11) = 3.30$, $p = 0.007$; Supplementary Figure 5B), and the amount of meditation training and change in respiration rate ($\chi^2(1) = 6.10$, $p = 0.014$; $t(7.68) = 2.47$, $p = 0.040$; Supplementary Figure 5C) but not alpha ($\chi^2(1) = 0.30$, $p = 0.58$), beta ($\chi^2(1) \sim 0$, $p = 0.96$), gamma ($\chi^2(1) = 0.48$, $p = 0.49$), heart-rate ($\chi^2(1) = 2.37$, $p = 0.12$) and heart-rate variability ($\chi^2(1) = 0.15$, $p = 0.70$). Correlation plots of the regression estimates for theta (Supplementary Figure 5B) and respiration rate (Supplementary Figure 5C) revealed that it was not highly trained LTMs that

increased these two NPMs over meditation blocks compared to BL, but rather the less trained LTMs who decreased theta and respiration rate compared to BL.

Neural and physiological differences between groups

Next, we performed exploratory analysis on how the neural and physiological measures differed between the groups and blocks. Because the two groups performed different meditation techniques during M1 and M2, we could not directly compare the effect of expertise on these blocks. Instead, we compared differences in NPMs between groups for BL and CH. We again used linear mixed models and regressed the group-by-block interaction and main effects on

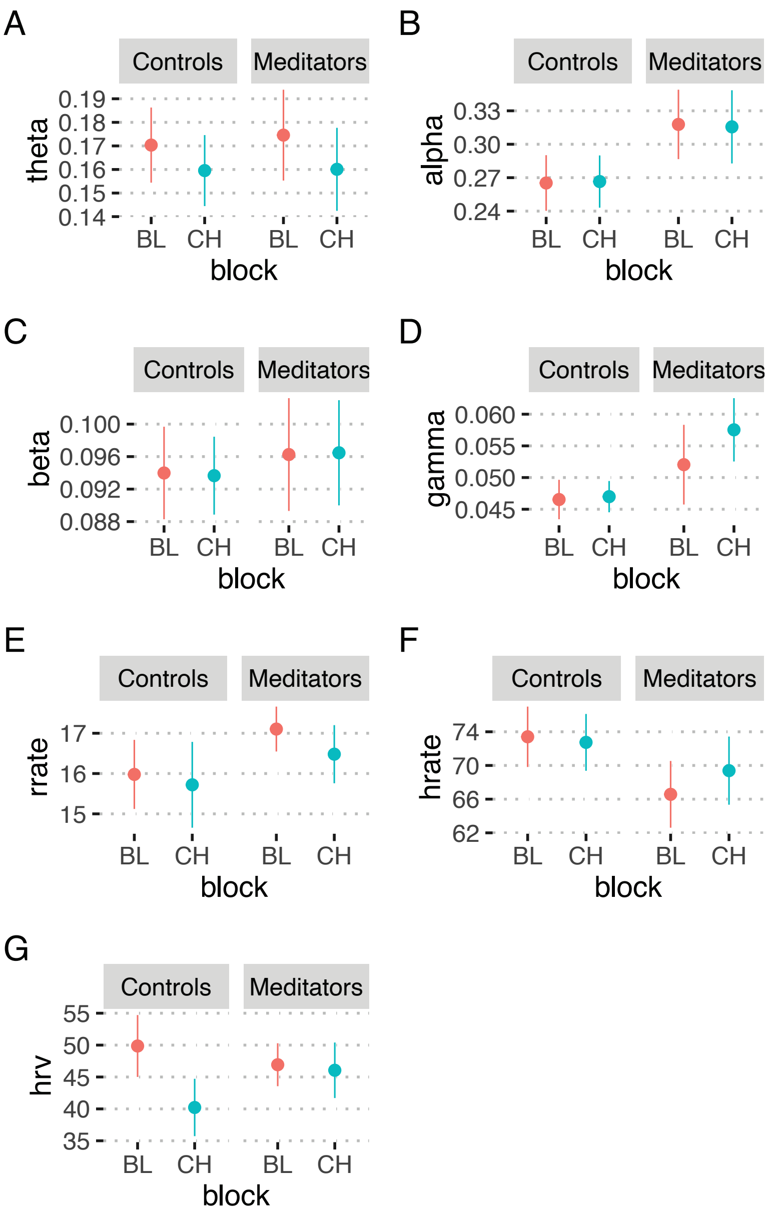

Supplementary Figure 6. Mean and standard errors of **A)** theta, **B)** alpha, **C)** beta, **D)** gamma amplitudes, and **E)** respiration rate, **F)** heart rate, and **G)** heart-rate variability for the two groups for the baseline and chanting blocks.

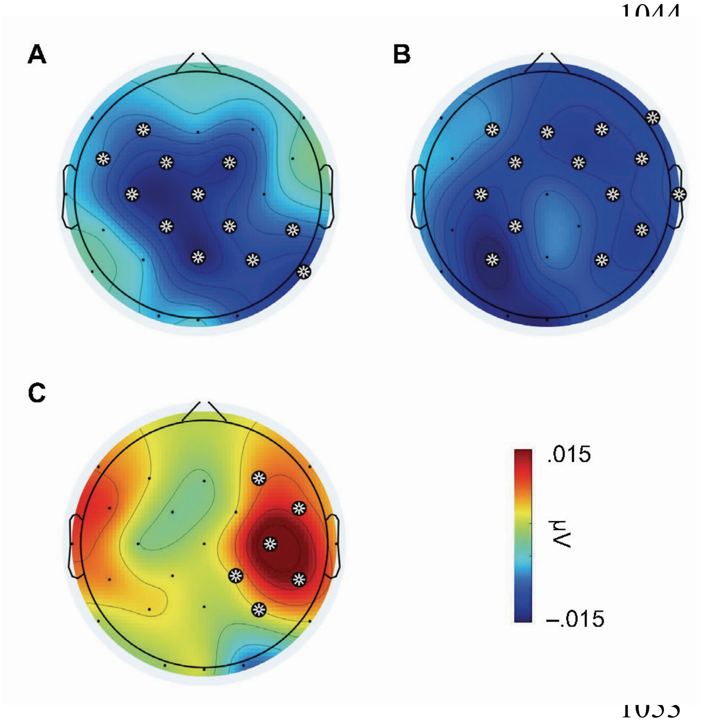

Supplementary Figure 7. Change in theta amplitude from BL to CH in **A)** LTMs and **B)** CTLs. **C)** Change in gamma amplitude from BL to CH in LTMs. Asterisks show scalp locations of channels that showed a significant difference $p < 0.05$ (Bonferroni-corrected for 26 channels).

the seven NPMs. Supplementary Table 1 provides the statistical values for the group-by-block comparison for seven NPMs, and Supplementary Figure 6 plots the changes in NPMs across groups and blocks.

There was a significant decrease in theta amplitude from BL to CH in both groups, which could be observed widely across the scalp (Supplementary Figure 7A–B). There was also an increase in gamma amplitude from BL to CH specifically in the LTMs, located over the right frontal and parietal channels (Supplementary Figure 7C). There was however no change in alpha or beta amplitude between the two groups and blocks. There was an increase in HR from BL to CH in the LTMs but not in the CTLs. There was also a trend across groups for a reduction in RR from BL to CH. Finally, HRV appeared to decrease from BL to CH in the CTLs but not in the LTMs.

Our analysis revealed no differences in BL between groups for any of the NPMs.

Supplementary Table 1. Statistical effects for comparing how the seven NPMs differed between groups and blocks (BL and CH only)

NPM	Interaction:	Main effect:	Main effect:	Post-hoc comparisons

	group x block		group		block		
	$\chi^2(1)$	p	$\chi^2(1)$	p	$\chi^2(1)$	p	
theta	0.71	0.40	0.01	0.91	38.93	<0.001	BL > CH<br>$t(341) = 6.24$; $p < 0.001$
alpha	0.22	0.64	1.70	0.19	0.00	0.96	
beta	0.09	0.76	0.09	0.76	0.03	0.87	
gamma	4.27	0.039					LTM:<br>CH > BL<br>$t(340) = 3.10$; $p = 0.002$<br>CTL:<br>CH > BL<br>$t(340) = 0.29$; $p = 0.78$
respiration rate	2.16	0.14	0.68	0.41	3.30	0.069	BL > CH<br>$t(249) = 1.81$; $p = 0.071$
heart rate	8.59	0.003					LTM:<br>CH > BL<br>$t(306) = 2.59$; $p = 0.010$<br>CTL:<br>CH > BL<br>$t(306) = –1.52$; $p = 0.13$
heart rate variability	2.99	0.084					LTM:<br>BL > CH<br>$t(198) = –0.45$; $p = 0.65$<br>CTL:<br>BL > CH<br>$t(190) = 2.11$; $p = 0.036$

Neural correlation with meditation depth over time

We used relatively long periods of meditation (15–20 mins) to enable participants to sufficiently enter deep states of meditation. As an exploratory analysis, we also examined how the alpha and theta correlations with meditation depth changed as a function of the duration of the meditation period. Supplementary Figure 8 shows the *Chi*-squared values of the 2-way interactions alpha x depth-level and theta x depth-level when regressed upon self-

reports separately for the two groups as a function of duration over which alpha and theta amplitudes were averaged starting from the beginning of a block. Filled triangles depict the time-points when the interactions were significant ($p < 0.05$). For CTLs, the meditation depth was significantly associated with alpha/theta over the entire range of 2–15 minutes starting from the beginning of the block. For LTMs, however, the significant association with both alpha and theta emerged in the 8–9-minute duration from the starting of the block and plateaued at ~12 minute into the block.

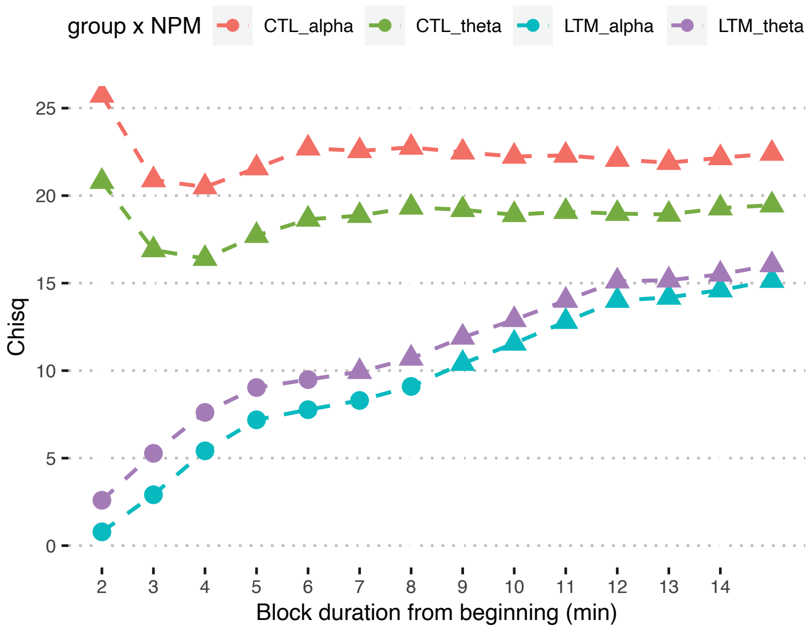

Supplementary Figure 8. Plot depicting how the association between meditation depth, and alpha and theta amplitude changed as a function of the time (in min) over which amplitude was calculated from the beginning of the meditation block separately for the two groups. Values plotted on the y-axis are the Chi-squared values of the interaction between depth-level, and alpha and theta amplitude when regressed using a linear mixed model upon self-reported ratings. Filled triangles are time points where the association was statistically significant ($p < 0.05$), while other points are shown by filled circles.

References

Anandamurti, S. S. (1998). *Ananda Marga Elementary Philosophy*. Kolkata, India: Ananda Marga Publications.

Anandamurti, S. S. (2010). *Yoga Sadhana*. Ananda Marga Publications.

Bharati, S. V. (2001). *Yoga Sutras of Patanjali: With the Exposition of Vyasa*. Motilal Banarsidass Publ.

Brandmeyer, T., & Delorme, A. (2016). Reduced mind wandering in experienced meditators and associated EEG correlates. *Experimental Brain Research*, 1–10. https://doi.org/10.1007/s00221-016-4811-5

Corby, J. C., Roth, W. T., Zarcone, V. P., & Kopell, B. S. (1978). Psychophysiological correlates of the practice of Tantric Yoga meditation. *Archives of General Psychiatry*, *35*(5), 571–577.

Delorme, A., & Makeig, S. (2004). EEGLAB: An open source toolbox for analysis of single-trial EEG dynamics including independent component analysis. *Journal of Neuroscience Methods*, *134*(1), 9–21. https://doi.org/10.1016/j.jneumeth.2003.10.009

Delorme, A., Sejnowski, T., & Makeig, S. (2007). Enhanced detection of artifacts in EEG data using higher-order statistics and independent component analysis. *NeuroImage*, *34*(4), 1443–1449. https://doi.org/10.1016/j.neuroimage.2006.11.004

Dor-Ziderman, Y., Ataria, Y., Fulder, S., Goldstein, A., & Berkovich-Ohana, A. (2016). Self-specific processing in the meditating brain: A MEG neurophenomenology study. *Neuroscience of Consciousness*, *2016*(1). https://doi.org/10.1093/nc/niw019

Dor-Ziderman, Y., Berkovich-Ohana, A., Glicksohn, J., & Goldstein, A. (2013). Mindfulness-induced selflessness: A MEG neurophenomenological study. *Frontiers in Human Neuroscience*, *7*. https://doi.org/10.3389/fnhum.2013.00582

Harper, K. A., & Brown, R. L. (2002). *The roots of Tantra*. Suny press.

Hewitson, J. M. (2014). Husserl's Epoché and Sarkar's Pratyáhára. *Comparative and*

Continental Philosophy, *6*(2), 158–177. https://doi.org/10.1179/1757063814Z.00000000039

Lumma, A.-L., Kok, B. E., & Singer, T. (2015). Is meditation always relaxing? Investigating heart rate, heart rate variability, experienced effort and likeability during training of three types of meditation. *International Journal of Psychophysiology*, *97*(1), 38–45. https://doi.org/10.1016/j.ijpsycho.2015.04.017

Taraka, & Acyutananda Avadhuta, A. (2014). *Ananda Marga: Social and Spiritual Practices* (3rd ed.). Ananda Marga Publications.

Travis, F., & Pearson, C. (2000). Pure Consciousness: Distinct Phenomenological and Physiological Correlates of "Consciousness Itself". *International Journal of Neuroscience*, *100*(1–4), 77–89. https://doi.org/10.3109/00207450008999678

van Lutterveld, R., Houlihan, S. D., Pal, P., Sacchet, M. D., McFarlane-Blake, C., Patel, P. R., Sullivan, J. S., Ossadtchi, A., Druker, S., Bauer, C., & Brewer, J. A. (2017). Source-space EEG neurofeedback links subjective experience with brain activity during effortless awareness meditation. *NeuroImage*, *151*, 117–127. https://doi.org/10.1016/j.neuroimage.2016.02.047

Vollmer, M. (2019). HRVTool – an Open-Source Matlab Toolbox for Analyzing Heart Rate Variability. *2019 Computing in Cardiology (CinC)*, Page 1-Page 4. https://doi.org/10.23919/CinC49843.2019.9005745
